# A systems pharmacology approach to identify the autophagy-inducing effects of Traditional Persian medicinal plants

**DOI:** 10.1038/s41598-020-79472-y

**Published:** 2021-01-11

**Authors:** Pouria Mosaddeghi, Mahboobeh Eslami, Mitra Farahmandnejad, Mahshad Akhavein, Ratin Ranjbarfarrokhi, Mohammadhossein Khorraminejad-Shirazi, Farbod Shahabinezhad, Mohammadjavad Taghipour, Mohammadreza Dorvash, Amirhossein Sakhteman, Mohammad M. Zarshenas, Navid Nezafat, Meysam Mobasheri, Younes Ghasemi

**Affiliations:** 1 Pharmaceutical Sciences Research Center, Shiraz University of Medical Sciences, Shiraz, Iran; 2 Department of Pharmaceutical Biotechnology, School of Pharmacy, Shiraz University of Medical Sciences, P.O. Box 71345-1583, Shiraz, Iran; 3 Student Research Committee, Shiraz University of Medical Sciences, Shiraz, Iran; 4 Cellular and Molecular Medicine Student Research Group, School of Medicine, Shiraz University of Medical Science, Shiraz, Iran; 5 Department of Medicinal Chemistry, School of Pharmacy, Shiraz University of Medical Sciences, Shiraz, Iran; 6 Institute of Biomedicine, University of Eastern Finland, Kuopio, Finland; 7 Department of Phytopharmaceuticals (Traditional Pharmacy), School of Pharmacy, Shiraz University of Medical Sciences, Shiraz, Iran; 8 Medicinal Plants Processing Research Center, Shiraz University of Medical Sciences, Shiraz, Iran; 9 Department of Biotechnology, Faculty of Advanced Sciences and Technology, Tehran Islamic Azad University of Medical Sciences, Tehran, Iran; 10 Iranian Institute of New Sciences (IINS), Tehran, Iran

**Keywords:** Computational biology and bioinformatics, Drug discovery, Systems biology, Molecular medicine

## Abstract

Aging is correlated with several complex diseases, including type 2 diabetes, neurodegeneration diseases, and cancer. Identifying the nature of this correlation and treatment of age-related diseases has been a major subject of both modern and traditional medicine. Traditional Persian Medicine (TPM) embodies many prescriptions for the treatment of ARDs. Given that autophagy plays a critical role in antiaging processes, the present study aimed to examine whether the documented effect of plants used in TPM might be relevant to the induction of autophagy? To this end, the TPM-based medicinal herbs used in the treatment of the ARDs were identified from modern and traditional references. The known phytochemicals of these plants were then examined against literature for evidence of having autophagy inducing effects. As a result, several plants were identified to have multiple active ingredients, which indeed regulate the autophagy or its upstream pathways. In addition, gene set enrichment analysis of the identified targets confirmed the collective contribution of the identified targets in autophagy regulating processes. Also, the protein–protein interaction (PPI) network of the targets was reconstructed. Network centrality analysis of the PPI network identified mTOR as the key network hub. Given the well-documented role of mTOR in inhibiting autophagy, our results hence support the hypothesis that the antiaging mechanism of TPM-based medicines might involve autophagy induction. Chemoinformatics study of the phytochemicals using docking and molecular dynamics simulation identified, among other compounds, the cyclo-trijuglone of *Juglans regia* L. as a potential ATP-competitive inhibitor of mTOR. Our results hence, provide a basis for the study of TPM-based prescriptions using modern tools in the quest for developing synergistic therapies for ARDs.

## Introduction

Aging is a progressive impairment that ultimately increases one’s vulnerability. Evidence suggests that inducing autophagy—an evolutionarily conserved pathway responsible for recycling the degraded proteins and organelles in the cell aimed at maintaining homeostasis— could prevent the occurrence, delay the progression and decrease the severity of several age-related diseases, such as neurodegenerative diseases, type 2 diabetes, cardiovascular diseases, and many others^1–3^. Autophagy, has a crucial role in reducing the age related pathological changes including accumulation of protein aggregates, inflammaging, cellular senescence and oncogenesis^4^.

Also, in most cases, longevity regulating pathways modulate autophagy. These include forkhead box O1 (FoxO), sirtuin 1 (SIRT1), AMP-activated protein kinase (AMPK), and mechanistic target of rapamycin (mTOR)^5–7^. In addition, autophagy inhibition, could circumvent the longevity-inducing effects of anti-aging agents such as rapamycin. So it seems that autophagy acts as the core process in longevity assurance mechanisms^8^.

Considering the complexity of human physiology, adopting a holistic approach capable of integrating different levels of physiological data is warranted. The currently available omics data, together with advances in systems biology tools and algorithms, enable such integrative approaches to treat human multifactorial health issues such as aging^9,10^.

Recent studies have discovered that combining anti-aging drugs have synergistic anti-aging benefits motivating further systems-oriented studies towards optimal aging treatment design^11,12^.

Given that autophagy is induced through different pathways, it can be assumed that the anti-aging effects of medicinal plants might be the result of various compounds present in the aforementioned plants, which can simultaneously induce autophagy via different pathways^13–15^.

Li first hypothesized the concept of the relationship between Traditional Chinese Medicine (TCM) syndromes, herbal formula, and molecular networks in 1999^16^. Since then, substantial advances in concepts and methodologies have been made to modernize TCM and turn it from an experience-based medicine into evidence-based medicine^17–21^. The methodologies of TCM systems pharmacology are mainly based on network construction to uncover the associations between network components and network analysis to identify critical components and functional modules in the network^22^. Several distinguished studies have been made and validated the proposed concepts and methodologies in this field^19,23,24^. For instance, Zhang et al*.* investigated the ancient concept in Traditional Chinese Medicine, ZHENG, have a molecular basis with regard to the neuro-endocrine-immune systems^25^.

Traditional Persian Medicine (TPM) represents a holistic temperament-dependent approach to human healthcare. According to TPM, illnesses are the result of an imbalance in temperament due to physiological, psychological, or external stimuli. However, apart from the validity of TPM's underlying assumption, it seems that the possible mechanism of herbal medicines is due to the interaction between pharmaceutical compounds with human proteins in a multi-compound/multi-target manner. This prompted us to conduct a systems pharmacological study to investigate the possible effects of herbal medicine in the treatment of ARDs^26–28^.

For this purpose, we searched the medicinal plants that are used to treat three or more ARDs, including cancer, cardiovascular diseases, neurodegeneration diseases, type 2 diabetes, and aging. Then, the phytochemicals of the selected medicinal plants were investigated in order to find their potential autophagy inducing action. Next, genes related to the mechanism of action of the natural products were used for enrichment analysis and network analysis. In addition, we proposed a novel natural product with the ability to inhibit mTOR, the major inhibitor of autophagy, using cheminformatics approaches.

## Method

### Identification of TPM plants relevant to ARDs therapies

**Figure 1:**
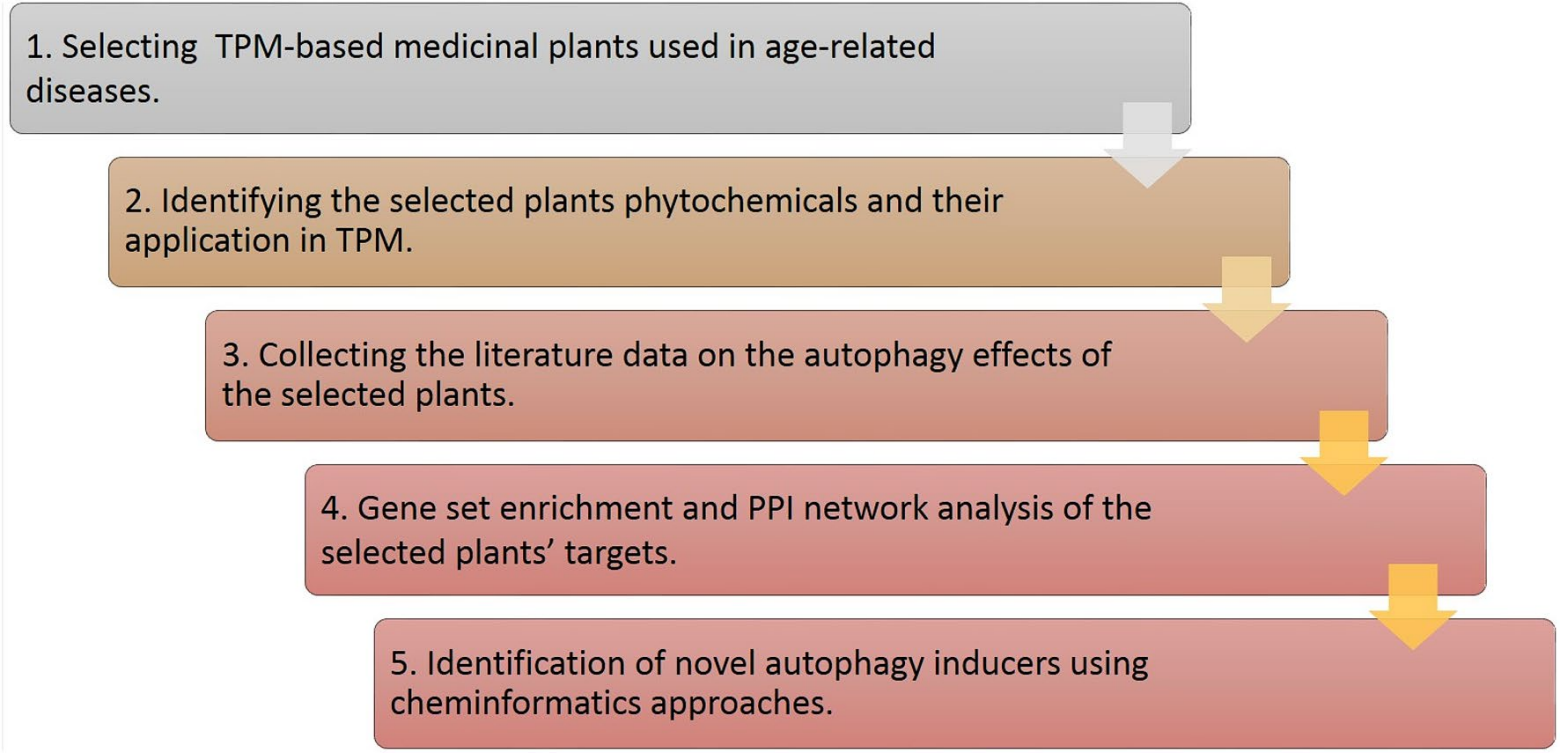
The workflow of this study.

In the first step of our study, the medicinal plants applied in the TPM-based treatment of ARDs, including cancer, cardiovascular diseases, neurodegeneration disorders, type 2 diabetes, and aging, were comprehensively reviewed^29–34^ to identify the relevant herbal medicines (Fig. 1). Noteworthy, the selected diseases are among the leading causes of death in the last years^35^.

Building on our previous work^29–32^and literature review)^33,34^, herbal medicines in TPM for ARDs were identified. A number of significant Persian medical and pharmaceutical manuscripts, such as *Liber Continents* by Rhazes^36^, The *Canon of Medicine* by Avicenna^37^, and *Tohfat ol Moemenin* by Mohammad Tunakabuni^38^ were used to retrieve geriatric remedies in these studies.

### Identification of TPM-related phytochemicals and their targets

After obtaining an extended catalog of TPM-related herbs, each of the herbs was submitted to Dr. Duke’s database (http://phytochem.nal.usda.gov) to identify the relevant phytochemicals. After removing the duplicate compounds, the name of each phytochemical was searched in the literature resources (PubMed and google scholar) together with the “autophagy” keyword to obtain the relevant academic articles. The obtained papers were then reviewed to retrieve the targets of the phytochemicals in question. Also, phytochemicals were classified based on the data retrieved from FooDB (www.foodb.ca). The obtained data on the TPM-based phytochemicals were then visualized.

### Gene set enrichment analysis

In order to obtain information regarding the targeted genes, enrichment analysis was done to compare the targets of phytochemicals with annotated gene sets. Consequently, genes related to the medicinal plants’ phytochemicals mechanism of action were enriched, using Enrichr^39,40^ method at http://amp.pharm.mssm.edu/Enrichr/. Gene ontology (GO)^41,42^ Biological Process (BP), GO Molecular Function, GO Cellular Component (CC) functional, and Kyoto encyclopedia of genes and genomes (KEGG) pathway^43,44^ enrichment analysis for the selected genes were performed via the above-mentioned tool.

### Protein–protein interactions and network reconstruction

In order to find the essential proteins and pathways in the medicinal plants’ targets, the protein–protein interaction network of the targets was extracted from STRING^45^ at https://string-db.org/. Then, with the help of Gephi^46^, the network was visualized. Also, different topological properties (eccentricity, closeness, radiality, betweenness, degree, stress, centroid, eigenvector, bridging) of the network were analyzed by Cytoscape^47^ plug-in, CentiScaPe^48^.

The results suggest that mTOR protein has a crucial role in the network. Hence, we used chemoinformatics approaches to find small molecules that can induce autophagy through mTOR inhibition.

### Compound-target interaction study by molecular docking

First, three 3D X-ray crystal structures of mTOR in complex with its inhibitors^49^ (4JSX, 4JT5, 4JT6) were retrieved from Protein Data Bank (http://www.rcsb.org). In order to prepare the 3D structure for docking, the co-crystallized ligands and water molecules were removed, and polar hydrogens were added using a bash script in Linux operating system. The ligand molecules were minimized and saved in ".pdbqt" format after adding partial charges, and ".pdbqt" file of ligands was prepared in AutoDock tools 1.5.6^50^. Self-docking simulations were performed with AutoDock Vina (1.1.2)^51^ within a docking box defined by the following parameters: size_x = 30, size_y = 30, size_z = 30 Å. The central atom of the co-crystallized ligands was selected as the center of the docking box in all self-docking simulations. Among the PDB structures, 4JSX-A (RMSD = 0.51) was chosen based on optimal self-docking criteria of RMSD < 2 as a validation protocol. The obtained grid box coordinates from the self-docking procedure for this PDB code (x = 53.482, center = 0.81, center = -46.68) was then used for the final docking of the small molecules.

The 3D structure of the phytochemicals (mol2 format), which were retrieved from Dr. Duke’s website, is required for further analysis. To this end, the open-source program, KNIME^52^, was used as a platform to perform automated requests on chemical websites, using their application programming interface. ChemSpider (http://chemspider.com) was used for converting chemical names into their formulas and other parameters of small molecules. To use it for big projects, it has set an API for which the users can download it as WSDL file and use their token in platforms such as KNIME. In KNIME’s space, first, a file reader was put to read the compounds’ names of a text file. Then a “Generic Web Service Client” with ChemSpider's token was used to search for these names. Another “Generic Web Service Client” was dedicated to obtaining the results, and another one was linked to get the following parameters: CSID, inCHI, inCHIkey, and SMILES. Then, Open Babel^53^ software was used to convert SMILES into 3D structure files. Finally, AutoDock tools 1.5.6^50^ was used to save ".pdbqts" from mol2 structures for the small molecules. All docking simulations were accordingly performed, and the binding energies of the poses were retrieved from output files.

Also, the SMILES codes of the phytochemicals were retrieved from the cactus database (at https://cactus.nci.nih.gov/) and the properties were calculated by RDkit^54^. In addition, a binary vector for each plant was compiled in which the presence or lack of the compounds were represented by 0 and 1. The resulting matrix was subjected to the hierarchical clustering analysis (HCA) method using Hamming distance.

### Molecular Dynamics (MD) Simulation of compound-target interactions

MD simulation is a suitable tool to study biological systems with respect to the molecular point of view. This technique helps protein and ligand molecules to acquire the best orientation relative to each other and establish the best interactions so that the desired complex moves toward a more stable situation.

Gromacs 5.0.7 was used to perform MD simulation on the selected complexes from the molecular docking process^55^. Most of the applied steps and conditions of MD simulation were similar to previous works, although there were slight changes. AMBER99SB-ILDN force field was applied to generate all necessary files for the protein molecule^56^. ACPYPE (or AnteChamber PYthon Parser interfacE) and the general amber force field were used to generate the topological descriptions of the ligands^57^. Each of the selected complexes was placed in the center of a cubic box and then filled with TIP3P model of water molecules. A minimal distance of 12 Å from any edge of the box and the periodic boundary conditions was applied. A Concentration of 150 mM of NaCl (physiological conditions) was added to neutralize the simulation system. Particle mesh Ewald (PME) and LINCS algorithm were used to treat long-range electrostatic interactions and constrain all bonds. The non-bonded cutoff of 10 Å was also used. In order to minimize the system, steepest descent algorithm was applied. After that equilibration under NVT ensemble at 300 K was conducted during 500 ps. NPT equilibration was subsequently applied at 1 bar pressure and 300 K temperature during 3000 ps. The velocity rescaling algorithm was used to control the temperature with a time constant 0.1 ps as the temperature coupling while Parrinello-Rahman barostat algorithm with a time constant of 1 ps was applied to control the pressure. Finally, MD production run was conducted for 50 ns under similar conditions at 1 bar and 300 K. Also, A time step of 2.0 fs was applied in all MD simulations.

### Calculation of protein–ligand binding free energies

Free energies calculations were performed using the MM/PBSA method^58,59^. GMXPBSA 2.1 was presented by Paissoni et al*.*^60^. The fundamental theory of this calculation was explained in detail in our previous publications^61,62^. In this study, the GROMACS trajectories were used to compute the affinity of the chosen ligands toward mTOR proteins by GMXPBSA 2.1. Water molecules were removed, and the calculations were conceded on a total of 200 frames.

## Results and discussion

### Phytochemicals of TPM plants relevant to ARDs treatment

Following our literature review, a total of 215 plants were identified to be practiced TPM for treatment of the ARDs^29–34^ (the entire list of all plants is represented in Supplementary Table S1 online). Among these, eight herbs that were found to be used in the treatment of at least three ARDs were selected for further inspection. The selected plants include *Allium sativum* L., *Juglans regia* L., *Santalum album* L., *Cinnamomum verum* J. Presl, *Terminalia chebula* Retz., *Coriandrum sativum* L., *Cocos nucifera* L., and *Cichorium intybus* L. The list of the phytochemicals of each plant and their properties, the resulting matrix of binary vector for each plant, and HCA analysis of the similarity of plants is provided in Supplementary Table S2_a-h online, Supplementary Table S3 online, and Fig. S1 online respectively. Also, the family, major chemical compositions^63^, parts that were used and their applications in TPM^64^ were reviewed in Supplementary Table S4 online. Review of modern academic literature confirmed that these herbs contain many compounds (63 out of 1078) that may exert anti-aging effects by inducting autophagy process, through targeting several signaling pathways^65–131^.

For instance, *Allium sativum* L. is documented in several reports ^132,133^ as an autophagy-inducing agent. In particular, Yung-Lin Chu et al*.* showed that the major component of the herb, i.e., allicin, could induce p53-mediated autophagy in Hep G2 cells^107^.

Besides, Poulus et al*.* showed that feeding mice with 6% or 9% *Juglans regia* L. (walnut) diet, can significantly reduce the accumulation of polyubiquitinated protein and activated autophagy^134^. Furthermore, the highest percentage of fatty acid in *Juglans regia* L. corresponds to linoleic acid, oleic acid, and palmitic acid. The effects of these phytochemicals in inducing autophagy were documented by different research groups^84,96,131,135^.

α-Santalol, which is one of the primary components of *Santalum album* L. (Indian sandalwood) oil, has a wide range of health benefits, including anti-cancer and anti-inflammatory effects^136^. It was shown that East Indian Sandalwood oil (EISO), which constitutes 45–50% of alpha-santalol, could induce autophagy^104^.

Also, the anti-aging effects of *Cinnamomum verum* J. Presl (cinnamon) is confirmed in various researches. Evidence shows that cinnamon could increase the life-span of model organisms^137–142^. Cinnamic aldehyde is the main compound of the bark oil of this medicinal plant. Chung, J. et al*.* showed that cinnamic aldehyde could induce autophagy in macrophages. Another study demonstrated that cinnamic aldehyde exerts a neuroprotective effect in a Parkinson’s disease model through autophagy inhibition^143^.

It is being known as the “king of medicines” in Tibet^144^, *Terminalia chebula* Retz. contains several natural products, such as punicalagin and geraniin, which have shown the ability to induce autophagy^92,144^. In regard to *Coriandrum sativum* L. (coriander), *Cocos nucifera* L. (Coconut), and *Cichorium intybus* L. (Chicory), the direct evidence on their inducing effect on autophagy is scarce. However, these plants contain several natural products with the known ability to induce autophagy^145–149^.

**Figure 2:**
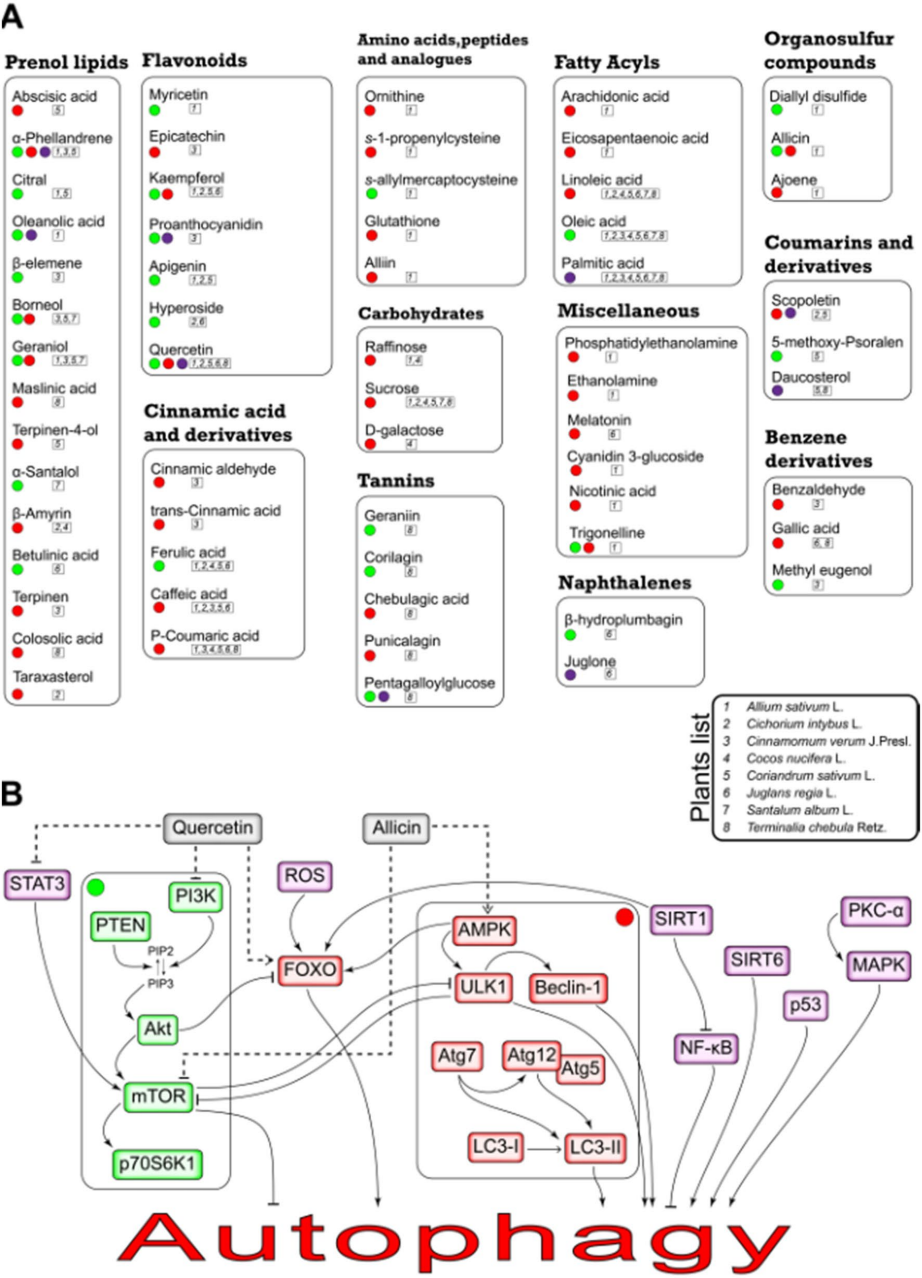
The schematic molecular mechanism of Autophagy-inducing effects of TPM-related phytochemicals (**A**) Each phytochemical comes with the number and colored circle, representing its related plant and mechanism of action, respectively. For instance, Allicin, a compound found in *Allium sativum* L., is represented by 1 and could induce autophagy by activating AMPK(red circle) and inhibiting mTOR(green circle). (**B**) Herbal medicines could modulate the key regulators of autophagy. The targets of phytochemicals were categorized into 3 groups (green, red, purple). The precise autophagy inducing effect of each phytochemical is also represented in Supplementary Table S5 online.

The activation/inhibition mechanisms through which the identified phytochemicals can influence autophagy is illustrated in Fig. 2. Also, the precise autophagy inducing effect of each phytochemical is represented in Supplementary Table S5 online.

### The targets of phytochemicals of the selected TPM plants

Through an extensive literate survey, we were able to link the phytochemicals of the selected herbs to 20 targets. The identified targets include protein kinase C alpha (PRKCA), autophagy-related 12(ATG12), beclin 1(BECN1), tumor protein p53(TP53), forkhead box O1(FOXO1), sirtuin 1(SIRT1), sirtuin 6(SIRT6), ribosomal protein S6 kinase B1(RPS6KB1), nuclear factor-kappa B subunit 1(NFKB1), phosphatase and tensin homolog(PTEN), signal transducer and activator of transcription 3(STAT3), AKT serine/threonine kinase 1(AKT1), mitogen-activated protein kinase 1(MAPK1), microtubule-associated protein 1 light chain 3 alpha(MAP1LC3A), autophagy-related 5(ATG5), unc-51 like autophagy activating kinase 1(ULK1), autophagy-related 7(ATG7), mitogen-activated protein kinase 8(MAPK8), protein kinase AMP-activated catalytic subunit alpha 1(PRKAA1), and mechanistic target of rapamycin (mTOR).

### Gene set enrichment analysis

As can be seen in Fig. 2, genes related to the medicinal plants’ chemical compounds mechanism of action were identified. (AKT1, ATG12, ATG5, ATG7, BECN1, FOXO1, MAP1LC3A, MAPK1, MAPK8, MTOR, NFKB1, PRKAA1, PRKCA, PTEN, RPS6KB1, SIRT1, SIRT6, STAT3, TP53, and ULK1).

**Figure 3:**
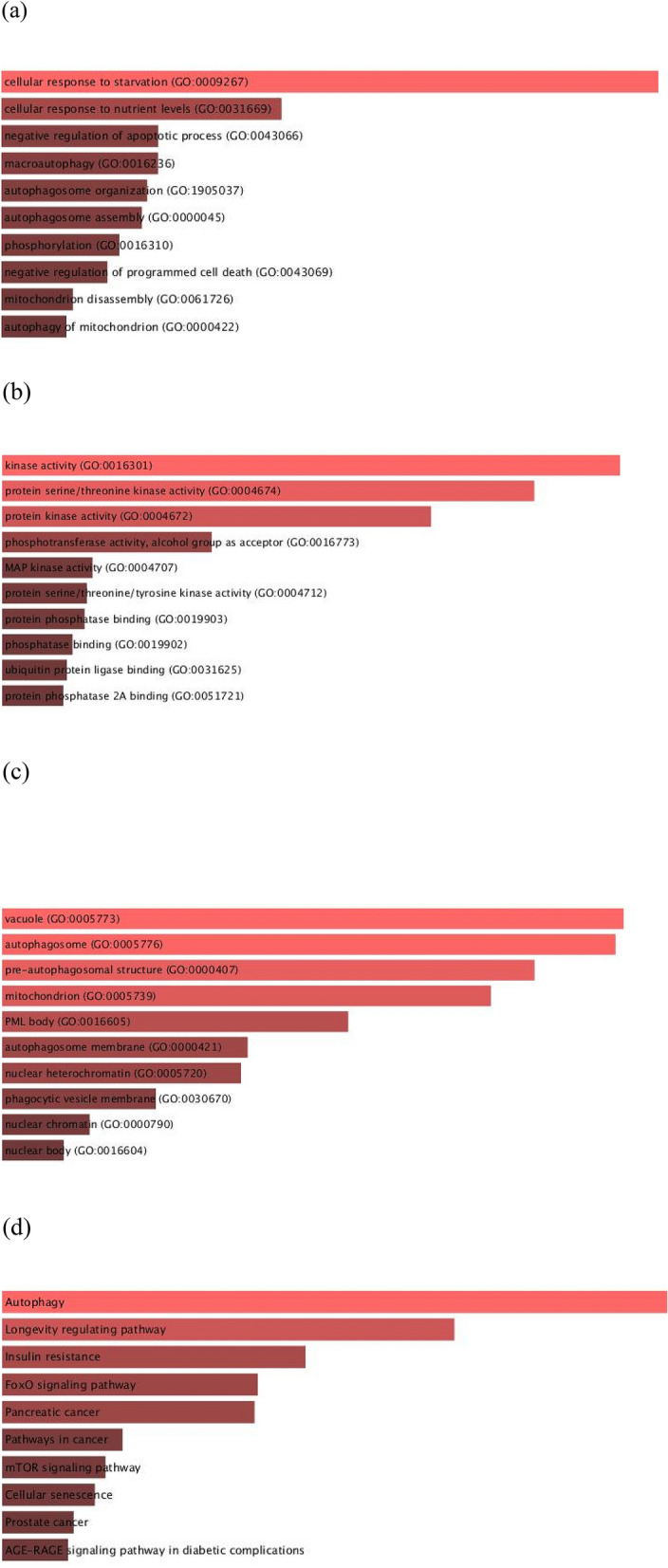
Gene ontology (GO) functional and Kyoto Encyclopedia of Genes and Genomes (KEGG) pathway enrichment analysis for the selected genes (performed via enricher). (**a**) GO Biological Process 2018; (**b**) GO Molecular Function 2018; (**c**) GO Cellular Component 2018; (**d**) KEGG 2019.

The enrichment analysis is a versatile method to gain insight into the pathways whose activity is influenced by a particular gene group. As seen in Fig. 3, our enrichment analysis indicated that the list of genes targeted by our medicinal plant compounds is most significantly associated with starvation, autophagy, and longevity regulating pathways. This provides a ground for traditional application of these plants in the context of ARDs.

### The PPI network of TPM phytochemicals’ targets

**Figure 4:**
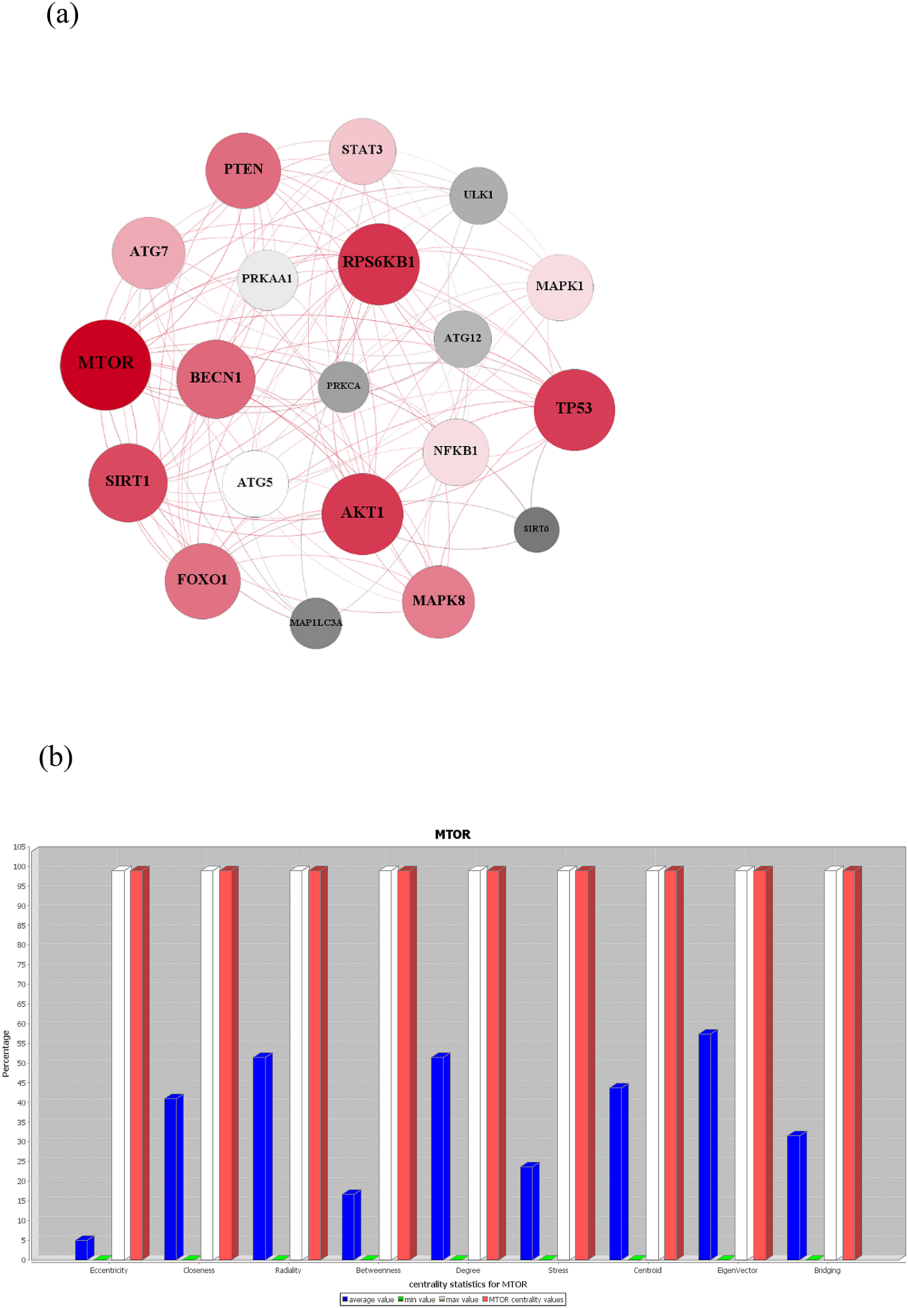
Network analysis of the medicinal plants’ targets. (**a**) Network visualization and analysis by Gephi. The size and the color of nodes represent the degree and eigenvector, respectively. (**b**) Network analysis of the medicinal plants’ targets. mTOR has a crucial role in this network since it has high centralities values for all the computed centralities.

We also constructed the protein–protein interaction network for the list of target genes (Fig. 4). Centrality analysis of the PPI network identified the network hub genes. Notably, mTOR was found to be the key hub in the PPI network (Fig. 4).

mTOR protein contributes to at least two distinct protein complexes: mTORC1(rapamycin sensitive) and mTORC2 (rapamycin-insensitive)^150,151^. mTORC1 is considered as a critical modulator of autophagy; most if not all autophagy inducing conditions, such as starvation, result from mTORC1 inhibition^14^. On the other hand, mTORC2 has a central role in cancer metabolic reprogramming. ATP-competitive mTOR kinase inhibitors (TKIs) like Torin2 _which can inhibit the catalytic domain of mTOR, and consequently, impede the function of both mTORC1 and mTORC2 _ has shown potent antitumor effects in the previous studies^152^. As such, we further used chemoinformatics tools to explore the potential of ATP-competitive mTOR inhibitors as a target for ARDs.

### Compound-target interaction validation via molecular docking

We further examined if the compounds of the TPM-based medicinal plants may indeed strongly inhibit mTOR. In doing so, the binding energy of 572 molecules in complex with mTOR protein were obtained by molecular docking (Supplementary Table S6 online). Amongst these molecules, two were selected as potential ATP-competitive inhibitors.

We considered Torin2, a well-known ATP competitive mTOR inhibitor, as a reference molecule, to evaluate the affinities of other ligands in question. According to the docking results (Supplementary Table S6 online), the binding energy of Torin2, cyclo-trijuglone, and sennoside-A in complex with mTOR were obtained −12.0, −12.6 and −9.6 kcal mol^−1^, respectively. Therefore, cyclo-trijuglone in complex with mTOR had the most favorable binding energy compared to Torin2, and sennoside A. 2D Structure of these molecules is shown in Supplementary Fig. S2 online. The mode of interaction with mTOR is shown in Supplementary Fig. S3 online. Ile2163, Pro2169, and Leu2185 and more importantly, Trp2239 are identified as crucial residues in mTOR-Torin2 complex^49^.

According to the docking results, Torin2 established π-π interaction with Trp2239. Furthermore, cation-π interaction and hydrogen bond were seen between Torin2 and Lys2187 and Val2240 residues, respectively. Similar to Torin2, cyclo-trijuglone molecule established π-π and cation-π interactions with Trp2239 and Lys2187, respectively. Moreover, sigma- π interaction was observed between this molecule and Met2345 residue. Hydrogen bonds were established between this molecule and Val2240 and Gly2238 residues. Trp2239 also interacted with sennoside A, through π-π stacking. Hydrogen bonds were also seen between this ligand and Asp2357, Gln2167, Ser2165, Ser2342, Lys2166, Vl2240, and Lys2171 residues. Many other residues also interacted with Torin2, cyclo-trijuglone and sennoside A, through electrostatic and hydrophobic interactions. While all studied ligands interacted with Trp2239, many other residues of this protein interacted with all three ligands.

### Molecular dynamics of compound-target interactions for selected phytochemicals

Molecular dynamics simulation can provide a broader landscape of how the compounds in the TPM-based medicinal plants may interact with their potential targets**.** Contrary to the docking, which assumes a rigid structure for protein, MD simulation allows the proteins residues to freely interact with the ligand and the complex reach to its most stable configuration. Therefore, MD allows for a more rigorous inspection of the extent of interaction between the ligand and the receptor. The selected complexes from molecular docking were used as the initial configuration in MD simulation, and the simulation product was run for 50 s.

**Figure 5:**
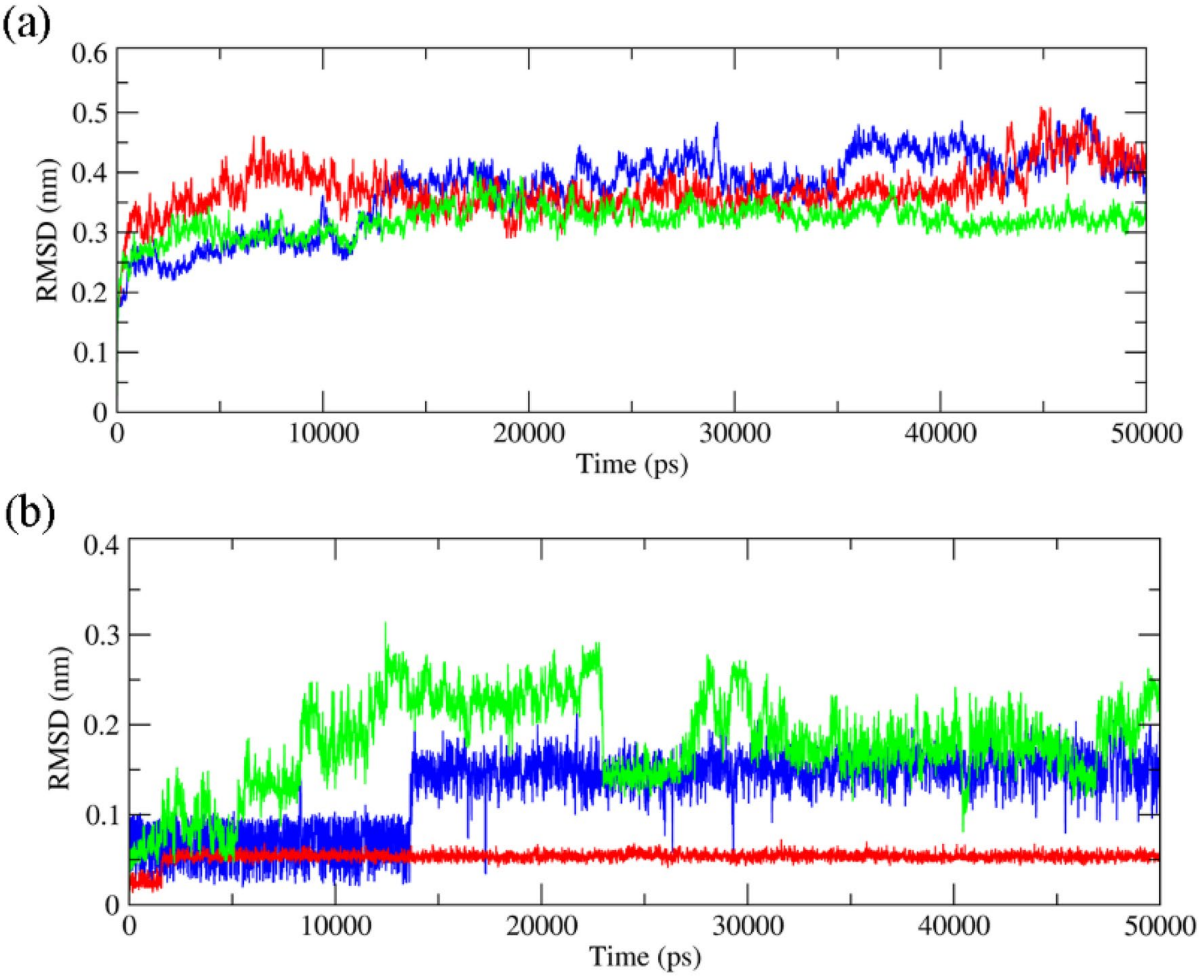
RMSD plots of the backbone atoms of the A-chain of mTOR protein and all three ligands. (**a**): Backbone RMSD plots of the A-chain of mTOR protein in complex with Torin2 (blue), cyclo trijoglone, and sennoside A (green), (**b**): RMSD plots of Torin2 (blue), cyclo trijoglone, and sennoside A (green) in complex with mTOR protein.

**Figure 6:**
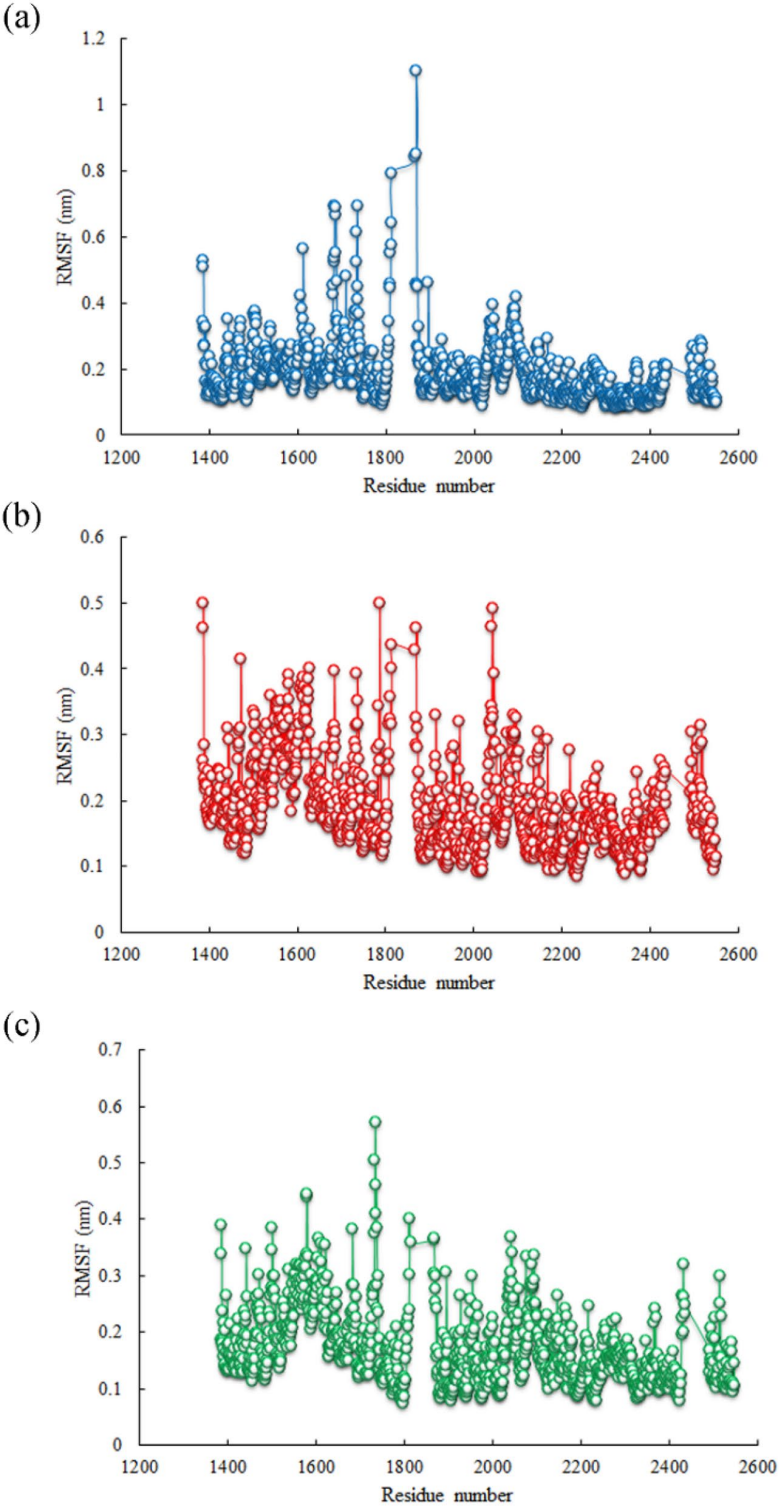
RMSF plots of all residues of the A-chain of mTOR protein in complex with (**a**) Torin2, (**b**) Cyclo trijoglone, (**c**) Sennoside A.

Firstly, Root Mean Square Deviation (RMSD) was evaluated for backbone atoms of the A-chain of mTOR protein and ligands. As seen in Figs. 5 and 6, the fluctuations of backbone atoms of mTOR protein in complex with sennoside A, cyclo trijoglone, and Torin2 molecules was increased. Since the RMSD parameter does not specify which region of the protein is more stable, the root mean square fluctuation (RMSF) parameter was evaluated for all residues of the A-chain of mTOR protein as well. This parameter identifies the most stable and unstable regions of a protein chain. As seen in Figs. 5 and 6, 1811–1867 residues and their neighbors tolerated significant fluctuation, especially in complex with Torin2. The mentioned residues are exposed to solvent, and they are located near the missing residues in the crystallographic structure; therefore, they generally endure more fluctuations in comparison with intra-residues. However, the missing residues and all residues with high fluctuations are distant from the active site of mTOR protein and cannot exert significant effects on protein–ligand interactions. On the other hand, sennoside A and cyclo-trijuglone have a larger structure than Torin2, so they interact with more residues of mTOR protein. These interactions affect the intramolecular interaction of a protein chain leading to a decrease in the fluctuations of some residues. As shown, the residues with higher fluctuations are seen in complex with Torin2 that has the smallest structure. As seen in the RMSD plots (Figs. 5, 6), sennoside A is more dynamics within the complex compared with Torin2 and cyclo-trijuglone, whereas Torin2 is the most stable within the mTOR pocket. The superimposition of the ligands at the final point of MD simulation over the obtained structure from the docking process confirmed that the ligands reached to their most stable configuration within the mTOR pocket. The obtained structures at the end of simulation time show that all three ligands could maintain their interaction with Trp2239 residue, although some interactions changed over the MD simulation period (Supplementary Fig. S4 online). The ligands modified their interactions with the essential residue of mTOR protein and implanted themselves within the active site of this protein through hydrogen bonds. As seen in Supplementary Fig. S5 online, the number of hydrogen bonds between all three ligands and mTOR protein increased during 50 ns MD simulation. Since sennoside, A molecule has more oxygen atoms than Torin2 and cyclo-trijuglone molecules, the number of hydrogen bonds between the former molecule and mTOR protein is higher than the other two ligands.

Finally, free energy calculation was performed to compare the affinity of all three ligands relative to mTOR protein, using GMXPBSA 2. The results (Table 1) revealed that the ability of Torin2 and cyclo-trijuglone molecules to inhibit mTOR protein was almost equal. Noteworthy, the binding energy values obtained from the docking study revealed that the affinity of Ciclo-trijoglone to mTOR protein was slightly more than Torin2.

**Table 1:** Binding free energies for protein–ligand complexes calculated with the MM/PBSA method for the MD simulations (kcal/mol).

Name of complex	Polar contribution		Non-polar contribution		Final value
	ΔE_coul_	ΔG_ps_	ΔE_vdw_	ΔG_nps_	ΔG_binding_
mTOR- Torin2	−27.7964	61.45839	−44.10026	−4.60590	−15.043947
mTOR- Cyclo trijuglone	−27.82053	70.06854	−51.86180	−5.14211	−14.82062
mTOR- Sennoside A	−39.461402	89.422709	−55.517817	−5.929588	−11.486338

These values showed that polar solvation contributions (ΔG_ps_) were unfavorable components in the complex formation while van der Waals (ΔG_vdw_), coulombic (ΔG_coul_), and non-polar solvation term (ΔG_nps_) components performed favorable roles in this regard.

Furthermore, the polar contributions, ΔE_coul_ and ΔG_ps_, revealed that the favorable component, electrostatic interaction, was not able to overcome the larger polar energy barrier. The non-polar contributions include Van der Waals interaction and the non-polar solvation effect. Embedding of hydrophobic fragments of the ligands within the active site of mTOR protein was done through mentioned forces; in this regard, the Van der Waals contribution performed a dominant role. Since the nature of the active site of mTOR protein is hydrophobic, the values of the ΔE_coul_ is fewer than the ΔE_vdw_ values. Sennoside A has many oxygen atoms, so ΔE_coul_ value of this molecule was more favorable in comparison with two other molecules; it also had the best ΔG_nps_ and ΔG_vdw_ values, but ΔG_ps_ value depredated all of these favorable components.

### Study limitations

Since autophagy is triggered via several pathways, many studies merely have uncovered the pathway that is induced/inhibited by the desired compound. Hence, we had to consider the name of the pathways as proteins in PPI-network. For instance, if a study showed that quercetin could trigger autophagy by inhibiting PI3K/AKT/mTOR signaling pathway, we considered PI3K, AKT, and mTOR as this compound’s targets.

In addition, we just searched the “phytochemicals’ names” that were retrieved from Dr. Duke’s website in combination with “autophagy.” Since some phytochemicals have synonyms, it is logical to assume that we missed autophagy-inducing phytochemicals that might have other names. Furthermore, a few phytochemicals were missed in the process of converting their names to 3D structures for further chemoinformatics analysis.

## Conclusions

The progressive advances in the field of systems biology and systems pharmacology underscore the ancient medicinal approaches such as Chinese and Persian traditional medicine as a valuable resource to address complex diseases. The present study was among the first to explore the molecular mechanism behind the TPM approach to the treatment of ARDs. According to our study, the plants used in TPM-based prescriptions contain many compounds with the potential to synergistically regulate the autophagy and its upstream pathways, providing a rationale for their documented antiaging effects. The consistency of TPM with chemical/biological data, as exemplified in the present study, provides motivation for future research into the TPM as an inspiring reference for the development of effective alternative medicine. Although further investigations should be performed to confirm the validity of our approach in vitro and in vivo.

## Supplementary Information

Supplementary information 1.

Supplementary information 2.

## Data Availability

All data generated and analyzed in this study are included in the main text or Supplementary Information files.
