# Author Correction: A systems pharmacology approach to identify the autophagy‑inducing effects of Traditional Persian medicinal plants

**DOI:** 10.1038/s41598-021-91771-6

**Published:** 2021-06-08

**Authors:** Pouria Mosaddeghi, Mahboobeh Eslami, Mitra Farahmandnejad, Mahshad Akhavein, Ratin Ranjbarfarrokhi, Mohammadhossein Khorraminejad‑Shirazi, Farbod Shahabinezhad, Mohammadjavad Taghipour, Mohammadreza Dorvash, Amirhossein Sakhteman, Mohammad M. Zarshenas, Navid Nezafat, Meysam Mobasheri, Younes Ghasemi

**Affiliations:** 1 Pharmaceutical Sciences Research Center, Shiraz University of Medical Sciences, Shiraz, Iran; 2 Department of Pharmaceutical Biotechnology, School of Pharmacy, Shiraz University of Medical Sciences, P.O. Box 71345‑1583, Shiraz, Iran; 3 Student Research Committee, Shiraz University of Medical Sciences, Shiraz, Iran; 4 Cellular and Molecular Medicine Student Research Group, School of Medicine, Shiraz University of Medical Science, Shiraz, Iran; 5 Department of Medicinal Chemistry, School of Pharmacy, Shiraz University of Medical Sciences, Shiraz, Iran; 6 Institute of Biomedicine, University of Eastern Finland, Kuopio, Finland; 7 Department of Phytopharmaceuticals (Traditional Pharmacy), School of Pharmacy, Shiraz University of Medical Sciences, Shiraz, Iran; 8 Medicinal Plants Processing Research Center, Shiraz University of Medical Sciences, Shiraz, Iran; 9 Department of Biotechnology, Faculty of Advanced Sciences and Technology, Tehran Islamic Azad University of Medical Sciences, Tehran, Iran; 10 Iranian Institute of New Sciences (IINS), Tehran, Iran

Correction to: *Scientific Reports* 10.1038/s41598-020-79472-y, published online 11 January 2021

The original version of this Article contained errors.

Affiliation 9 was incorrectly given as “Department of Biotechnology, Faculty of Advanced Sciences and Technology, Tehran Medical Sciences, Islamic Azad University, Tehran, Iran.”

The correct affiliation is listed below:

Department of Biotechnology, Faculty of Advanced Sciences and Technology, Tehran Islamic Azad University of Medical Sciences, Tehran, Iran

Additionally, an affiliation for author Meysam Mobasheri was omitted. The correct affiliation is listed below as affiliation 10.

Iranian Institute of New Sciences (IINS), Tehran, Iran.

The original Article and accompanying Supplementary Information file have been corrected.

