# Supplementary material for: A systems pharmacology approach to identify the autophagy-inducing effects of Traditional Persian medicinal plants
Source: Sci Rep. 2021 Jan 11;11:336. doi: 10.1038/s41598-020-79472-y (PMC7801619; doi:10.1038/s41598-020-79472-y)
Supplement: Supplementary file 1 — Supplementary information 1. [file 41598_2020_79472_MOESM1_ESM.docx]

**A Systems Pharmacology Approach to Identify the Autophagy-Inducing Effects of Traditional Persian Medicinal Plants**

Pouria Mosaddeghi^1,2,3,4^, Mahboobeh Eslami^1,2^, Mitra Farahmandnejad^1,2,3,4^, Mahshad Akhavein^1,2,3,4^, Ratin Ranjbarfarrokhi^1,2,3,4^, MohammadHossein Khorraminejad-Shirazi ^3,4^, Farbod Shahabinezhad^3,4^, Mohammadjavad Taghipour^1,2,3,4^, Mohammadreza Dorvash^1,3,4^, Amirhossein Sakhteman^5,6^, Mohammad M. Zarshenas^7,8^, Navid Nezafat^1,2^, Meysam Mobasheri^9,10^, Younes Ghasemi^1,2^


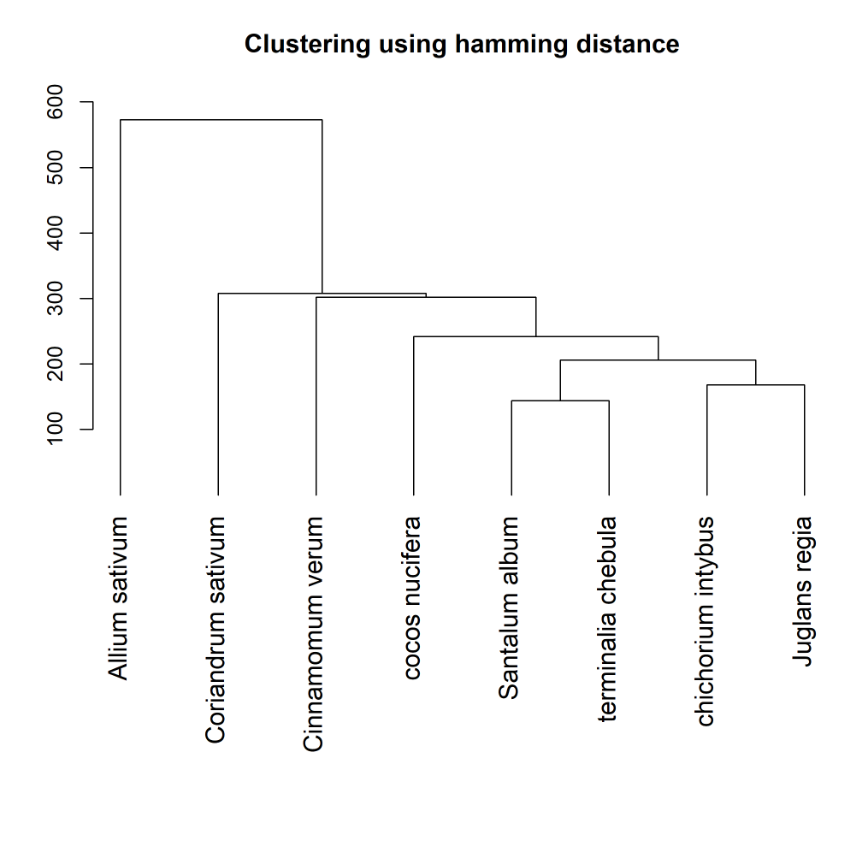


Supplementary Fig. S1: The similarity of the selected plants based on the hierarchical clustering analysis (HCA) method.


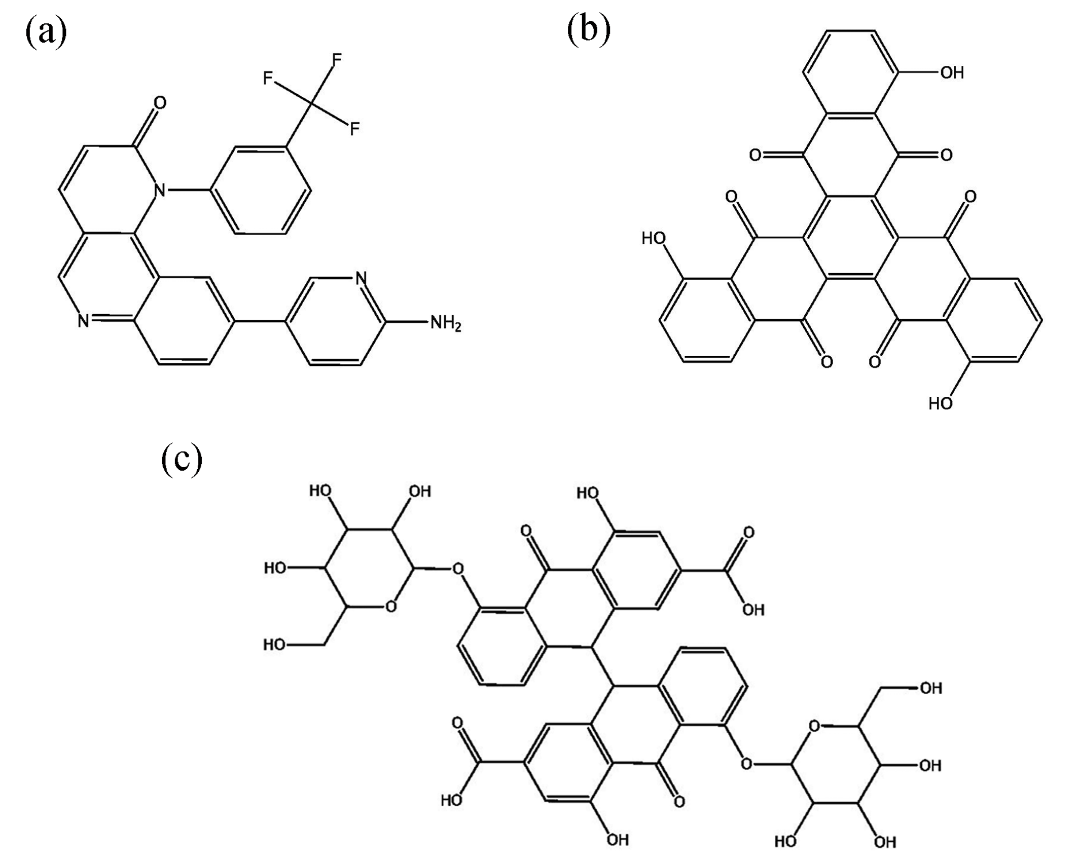


Supplementary Fig. S2: 2D Structure of three desired molecules; (a): Torin2 (b) Cyclo-trijuglone, (c) sennoside-A


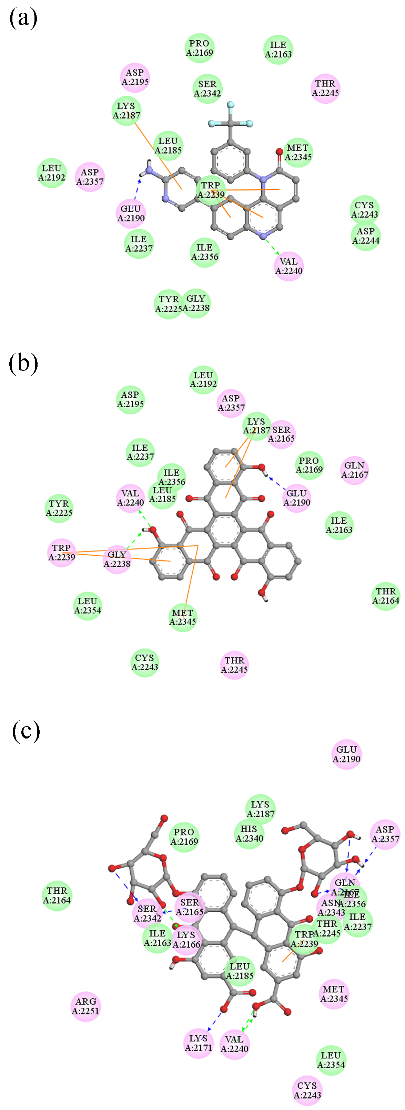


Supplementary Fig. S3**:** Two-dimensional representations of the observed interactions between the A-chain of mTOR protein and all three ligands after docking procedure; residues in a green frame: van der Waals interaction, residues in a magenta frame: electrostatic interaction, Orange lines: π-interactions, blue dashed line with an arrow at H-bond acceptor: hydrogen bond with amino acid side-chain, green dashed line with an arrow at H-bond acceptor: hydrogen bond with amino acid main chain; (a) mTOR in complex with Torin2, (b) mTOR in complex with Cyclo trijoglone, (c) mTOR in complex with Sennoside A.


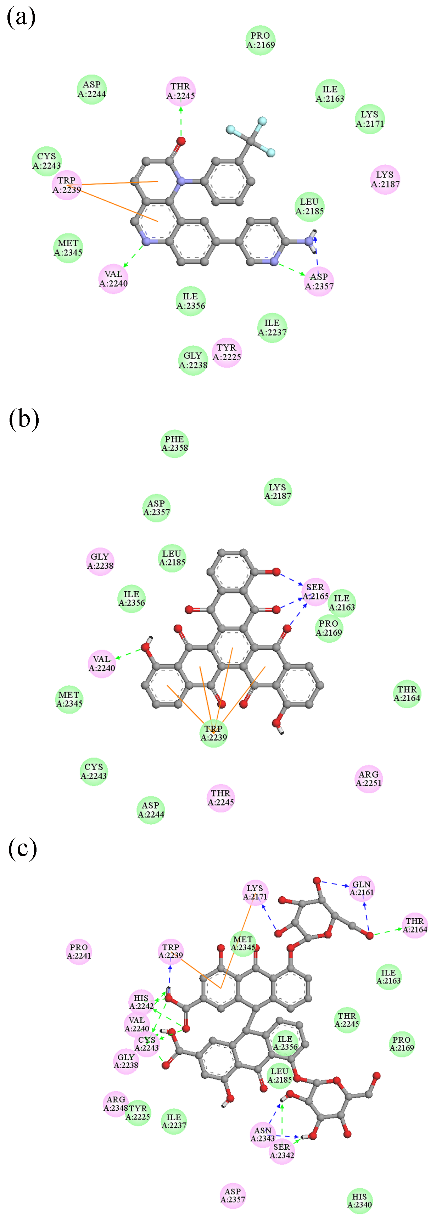


Supplementary Fig. S4**:** Two-dimensional representations of the observed interactions between mTOR protein and all three ligands after MD simulation; residues in a green frame: van der Waals interaction, residues in a magenta frame: electrostatic interaction, Orange lines: π-interactions, blue dashed line with arrow at H-bond acceptor: hydrogen bond with amino acid side-chain, green dashed line with arrow at H-bond acceptor: hydrogen bond with amino acid main chain; (a) mTOR in complex with Torin2, (b) mTOR in complex with Cyclo trijoglone, (c) mTOR in complex with Sennoside A.


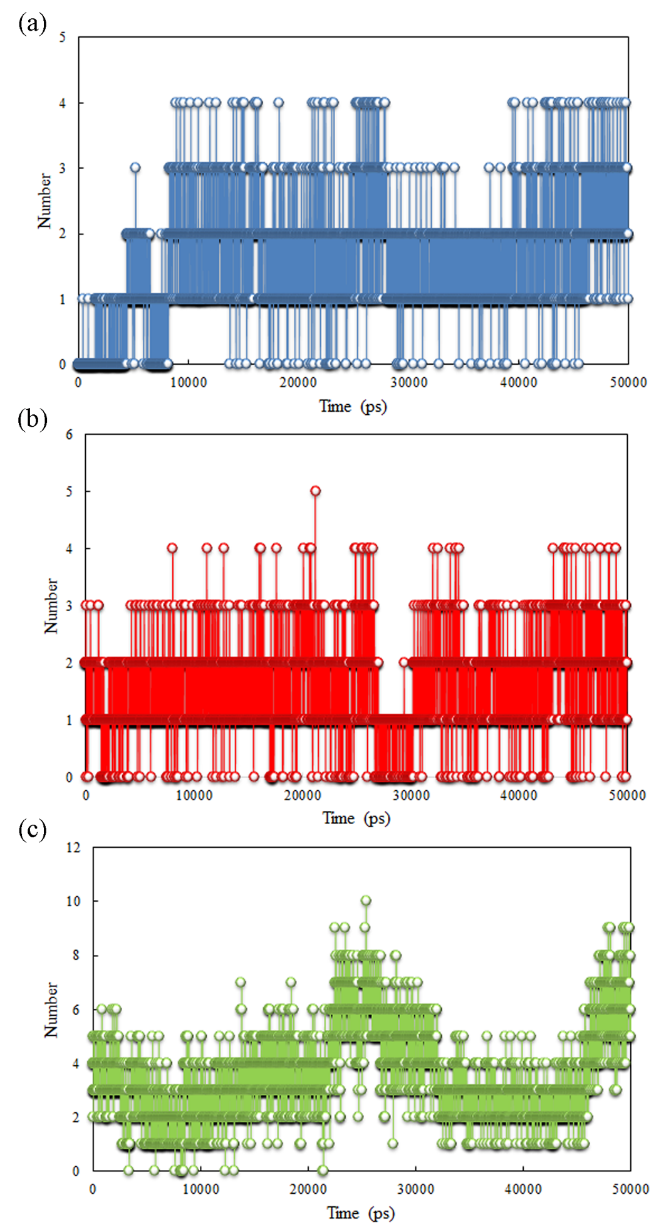


Supplementary Fig. S5**:** The number of hydrogen bonds between the A-chain of mTOR protein and: (a) Torin2, (b) Cyclo trijoglone, (c) Sennoside A.
